# *Baccharis trimera* Infusion Reduces Macrophages Activation and High-Fat Diet-Induced Metabolic Disorders in Mice

**DOI:** 10.3390/ph15101258

**Published:** 2022-10-13

**Authors:** Thalita Vieira Nascimento Ximenes, Raquel Carvalho, Iluska Senna Bonfá, Vanessa Samúdio Santos, Luciane Candeloro, Flávio Macedo Alves, Denise Brentan Silva, Carlos Alexandre Carollo, Karine de Cássia Freitas Gielow, Saulo Euclides Silva-Filho, Mônica Cristina Toffoli-Kadri

**Affiliations:** 1 Pharmaceutical Sciences, Food and Nutrition College, Federal University of Mato Grosso do Sul, Campo Grande 79070-900, Brazil; 2 Biosciences Institute, Federal University of Mato Grosso do Sul, Campo Grande 79070-900, Brazil

**Keywords:** obesity, hyperlipidic diet, inflammation, Swiss mice

## Abstract

The aim of this study is to evaluate the efficacy of *Baccharis trimera* infusion on high-fat diet-induced metabolic disorders in mice and macrophages activation. This study evaluated obesity, insulin resistance, dyslipidemia and hepatic steatosis induced by a high-fat diet in Swiss mice. Cellular parameters in macrophages, such as cell viability (MTT), the production and release of nitric oxide (NO) and hydrogen peroxide (H_2_O_2_), cell spreading, cell adhesion and phagocytosis were determined. Our results showed that treatment with *B. trimera* prevented the mentioned conditions, except for the production of hydrogen peroxide. *B. trimera* prevented the development of obesity and associated comorbidities, as well as activation of macrophages. In conclusion, *B. trimera* is able to prevent obesity and metabolic disorders and macrophages activation, minimizing inflammation and validating the popular use of this plant tea.

## 1. Introduction

Medicinal plants are particularly relevant because they can be used as complementary therapy to prevent diseases as well as a source of potential drug prototypes [1]. *Baccharis trimera* is a species recommended by the Brazilian Pharmacopoeia by having medicinal properties and recognized by the regulatory health agency [2]. The *Baccharis* genus, belonging to the Asteraceae family, comprises more than 500 species, distributed in the American continent and predominant in Argentina, Colombia, Chile, Mexico and Brazil [3]. This plant is popularly known as *carqueja*, and has several therapeutic purposes described in the literature, such as hepatoprotective [4,5], antiulcerogenic [6], anti-inflammatory [7], antioxidant and antiadipogenic properties [8,9].

Chronic noncommunicable diseases are long-term multifactorial diseases that develop over the course of life, and are triggered mainly by poor diet, physical inactivity, smoking and alcohol. Among these, obesity is a multifactorial clinical condition resulting from the interaction of genetic and environmental factors. Overweight is defined as excessive accumulation of body fat, mainly in the visceral adipose tissue, being one of the major factors for the emergence of comorbidities associated with obesity, as metabolic syndrome [10].

Metabolic stress responses to chronic caloric excess and cell death, may trigger inflammatory response in the liver, intestine and adipose tissue [11,12]. In this process, the adipocytes become hypoperfused, triggering the release of inflammatory mediators and the recruitment of macrophages to adipose tissue [13]. The macrophages are activated and enhance the inflammatory process in the tissue, triggering chronic, systemic and low-intensity inflammation [14]. This inflammatory condition is related to the appearance of metabolic disorders such as diabetes mellitus, hyperlipidemia [15], arthritis, arterial, atherogenesis and, consequently, the metabolic syndrome [16].

Although *B. trimera* is commonly used by the population, studies that evaluated the effects of this plant on cellular functions of macrophages under inflammatory conditions associated with obesity are insufficient. Therefore, the aim of this study was to evaluate the efficacy of *B. trimera* on high-fat diet-induced metabolic parameters in mice and macrophages activation with the perspective of contributing to the treatment of obesity and its comorbidities.

## 2. Results

### 2.1. Chemical Profile of the BTi

High-performance liquid chromatography coupled to mass spectrophotometry of the BTi showed the presence of 15 compounds (Figure S1, Supplementary Material) distributed in three main classes, caffeic acid derivatives (peaks 1, 2, 3, 8, 9, 10 and 13), flavonoid derivatives (peaks 4, 5, 6, 7 and 15) and diterpenoids of the clerodane type (peaks 11 and 12) (Table 1).

The compounds quinic acid (peak 1), caffeoylquinic acid (also called chlorogenic acid) (peak 2), rutin (peak 7), 3,4-dicaffeoylquinic acid (peak 8), 3,5-dicaffeoylquinic acid (peak 9), and 4,5-dicaffeoylquinic acid (Peak 10) had their identification confirmed by comparison with authentic standards.

Peak 3 *m*/*z* 367.1035 (C_17_H_19_O_9_)^−^ showed a UV spectrum compatible with caffeic acid, and the *m*/*z* 191 fragment was compatible with 5-feruloylquinic acid [17]. This compound has already been reported in specie of *B. trimera* in a survey of their chemical profiles performed by de Araújo et al. (2017) [18].

Peaks 4, 5 and 6 showed an *m*/*z* 563.1406 (C_26_H_27_O_14_)^−^ and a fragmentation pattern characteristic of C-glycosylated flavonoids [19]. Peaks 4 and 5 exhibited the fragment *m*/*z* 503 [M-H-_60_]^−^, indicating a pentose at position 6, which was not observed for compound 6. Zhou et al. (2012) [19] determined the order of retention and fragmentation profile of the flavonoid C-glycosides vicenin 1, isoschaftosid, and schaftosid, among others. Our data are in agreement with those observed, where vicenin 1 exhibited minor retention time and the presence of the fragment [MH-60]^−^, followed by isoschaftosid and finally schaftosid, which did not present the fragmentation pattern related to the pentose in position 6. Isoschaftosid has already been reported in *Baccharis* [20].

Peaks 11 (C_20_H_26_O_5_) and 12 (C_20_H_28_O_5_) were identified as diterpenoids of the clerodane type. These compounds did not adsorb UV radiation and exhibited the formation of adducts with formic acid in the negative mode. The literature has demonstrated the occurrence of this class in *B. trimera* [21]. Despite this previous occurrence, the lack of fragmentation studies prevented the structural determination of the compounds.

Peak 13 showed a caffeic acid with compatible UV spectra and fragments in the *m*/*z* 679 (C_36_H_55_O_12_)^−^ relative to the loss of a phenylpropanoid, in addition to the *m*/*z* 179 (C_9_H_7_O_4_)^−^/161 (C_9_H_5_O_3_)^−^ that characterized the presence of this fragment. Molecular weight, retention time and fragment data allow us to putatively determine this compound as a triterpene bound to a hexose and a caffeic acid.

The mass spectrum for peak 15 showed an *m*/*z* 343.0822 (C_18_H_15_O_7_)^−^ identified as dihydroxy-trimethoxyflavone. This compound has been reported in *B. trimera*, and its *m*/*z* 313 and 285 fragments are in agreement with the literature data for this compound [18].

### 2.2. Effect of the BTi on Food and Caloric Intake, Weight, Organ and Adipose Cushion Weights, and the Adiposity Index

As shown in Table 2, daily feed intake was lower in the groups receiving HD than in the groups fed ND. Regardless of the diet, the BTi animals had reduced feed intake compared to those treated with water. In addition, even when consuming less diet, both groups fed HD ingested more kilocalories per day than those fed ND. Regarding the weight of the animals, all groups started with equivalent values and after eight weeks, the final weight of the HD + water group was significantly higher than those of the other groups. It is worth mentioning that the HD + BTi group presented a final weight statistically similar to that of the ND + water group. Regardless of diet, the BTi groups had reduced final weights of the animals when compared to the corresponding groups treated with water.

The HD + water group presented greater weight gain than the other groups. However, regardless of diet, the BTi reduced the weight gain of the animals in the BTi groups compared to those in the groups treated with water. There was no significant difference between the weight gains of the ND + water and HD + BTi groups in the experimental period.

HD increased the weight of the visceral adipose cushions compared to ND, and BTi reduced the weight of the adipose retroperitoneal and mesenteric cushions of the animals that ingested HD when compared to water treatment. There was a difference in the adipocyte area of all groups evaluated. BTi reduced the area of the adipocytes from both groups, regardless of diet, compared to those treated with water.

Regarding the organs, the liver of the HD + water group presented greater weight than those of the other groups. It should be noted that the liver weight of the HD+ BTi group remained similar to that of the ND + water group. Both the diet and BTi did not change the weight of the other evaluated organs (data not shown).

When we analyzed the weight of the visceral adipose tissue, we observed that the HD + water group presented a greater adipose mass gain than the other groups and that the BTi group prevented the excessive gain of adipose mass by the animals in the HD + BTi group in comparison to those in the HD + water group. It is noteworthy that the adipose mass gain of the HD + BTi group was similar to that of the ND + water group.

The adiposity index of the animals showed the same pattern of response as that observed in the variations in the body mass gain, corroborating this result. Thus, we verified that the HD induced obesity in water-treated animals when compared to the ND. On the other hand, the HD + BTi group did not develop obesity, since there was no difference between the adiposity index of this group and the ND + water group. In addition, the BTi reduced the rate of adiposity of the animals, regardless of diet.

### 2.3. Effect of the BTi on the Glycemic Profile

**Figure 1:**
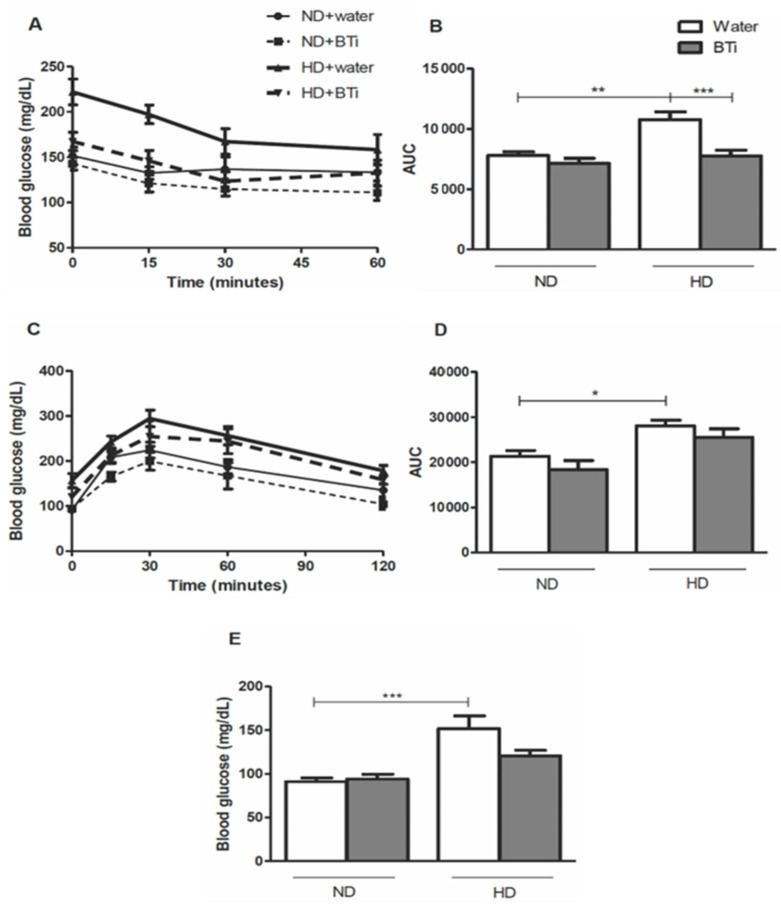
Effect of BTi on the glycemic profile of animals. (**A**) Glycemia values for Insulin sensitivity curve, according to the experimental group, at each time. (**B**) Area under the insulin sensitivity curve, according to the experimental group. (**C**) Glycemia values for oral glucose tolerance curve, according to the experimental group, at each time. (**D**) Area under the oral glucose tolerance curve, according to the experimental group. (**E**) Fasting glycemia. Results represents mean ± S.E.M. *n* = 8 animals/group. ND: normolipid diet. HD: high-fat diet. BTi: infusion of *B. trimera*. * *p* < 0.05. ** *p* < 0.01. *** *p* < 0.001. ANOVA, followed by Tukey post-test.

The effect of the BTi on insulin sensitivity, oral glucose tolerance test (OGTT) results and fasting glycemia are summarized in Figure 1. At time zero, prior to the intraperitoneal administration of insulin, the HD + water group had higher glycemia (222.00 ± 14.27 mg/dL) than the ND + water group (151.50 ± 9.57 mg/dL), ND + BTi group (142.75 ± 6.96 mg/dL) (*p* < 0.001) and the HD + BTi group (167.22 ± 10.12 mg/dL) (*p* < 0.01). This response profile was maintained 15 min after the insulin injection (ND + water, 132. 50 ± 6.84 mg/dL; ND + BTi, 120.88 ± 9.35 mg/dL; and HD + 145.89 ± 11.46 mg/dL), and 30 min after, the HD + BTi group had lower glycemia (123.44 ± 10.26 mg/dL) than the HD + water group (167.29 ± 14.07 mg/dL) (*p* < 0.05). One hour after the insulin injection, there were no differences in blood glucose between any of the groups analyzed (ND + water, 133.25 ± 9.26 mg/dL; ND + BTi, 110.88 ± 8.56 mg/dL; HD + water, 158.29 ± 16.87 mg/dL; and HD + BTi, 132.44 ± 14.36 mg/dL) (*p* > 0.05) (Figure 1A). The area under the curve (AUC) revealed that the HD + water group became insulin resistant (10,763 ± 640 AUC) when compared to the ND + water group (7784 ± 312 AUC). In contrast, the HD + BTi group did not develop insulin resistance (7759 ± 477 AUC), since its area under the curve in the insulin sensitivity test was similar to that of the ND + water group (7784 ± 312 AUC), as demonstrated in Figure 1B. There was no difference between the glycemic curves in response to insulin injection in the ND + water group (7784 ± 312 AUC) compared to the ND + BTi group (7129 ± 426 AUC) (*p* > 0.05). Likewise, the AUC of the OGTT showed that the HD + water group developed hyperglycemia (27,059 ± 2317 AUC) when compared to the ND + water (17,370 ± 1435 AUC) and ND + BTi (13,382 ± 1410 AUC) groups (Figure 1D). In contrast, a glycemia modulating effect was observed in the HD + BTi group, since there was no difference between the AUC of this group (25,530 ± 1849 AUC) and that of the ND + water group (21,308 ± 1267 AUC) (*p* > 0.05).

In relation to the fasting glycemia of the animals, the HD + water group presented a state of hyperglycemia (151.8 ± 14.68 mg/dL) when compared to the ND + water group (91.11 ± 4.38 mg/dL) and the ND + BTi group (94.00 ± 5.60 mg/dL), as shown in Figure 1E. In contrast, the BTi modulated the hyperglycemia generated by the HD, since there was no difference between the glycemia of the HD + BTi group (120.5 ± 6.48 mg/dL) and that of the ND + water group (*p* > 0.05). In addition, as in the insulin sensitivity test, the BTi did not alter the fasting glycemia of animals fed the ND and ND + BTi (*p* > 0.05).

### 2.4. Effect of the BTi on the Lipid Profile

Neither diet nor treatment altered the serum triglyceride (TG) level in any of the groups studied. The same was observed for the levels of high-density cholesterol (HDL-c) and very low-density cholesterol (VLDL). The HD + water group showed a significant increase in the serum level of low-density cholesterol (LDL-c) compared with the ND + water and ND + BTi groups, as observed in Table 3. It was observed that the BTi prevented the increase in LDL-c levels in the animals fed the HD when compared to water injection. Furthermore, the BTi maintained the serum LDL-C level of the HD + BTi group at a concentration similar to those of the ND + water and ND + BTi groups. Furthermore, we observed that the HD was effective for the development of hypercholesterolemia when compared to the ND. However, the BTi reduced the serum level of CT in the HD + BTi group compared to the HD + water group, in addition to maintaining this parameter at a level similar to that of the ND + water group.

When we calculated the atherogenic index (IA) of the animals, we observed that these results corroborate those found for the serum levels of TC and LDL-c. The HD + water group had a higher atherogenic index than both ND groups. In contrast, the atherogenic index of the HD + BTi group was significantly lower than that of the HD + water group and similar to those of the ND + water and ND + BTi groups.

### 2.5. Effect of the BTi on Histological Parameters

**Figure 2:**
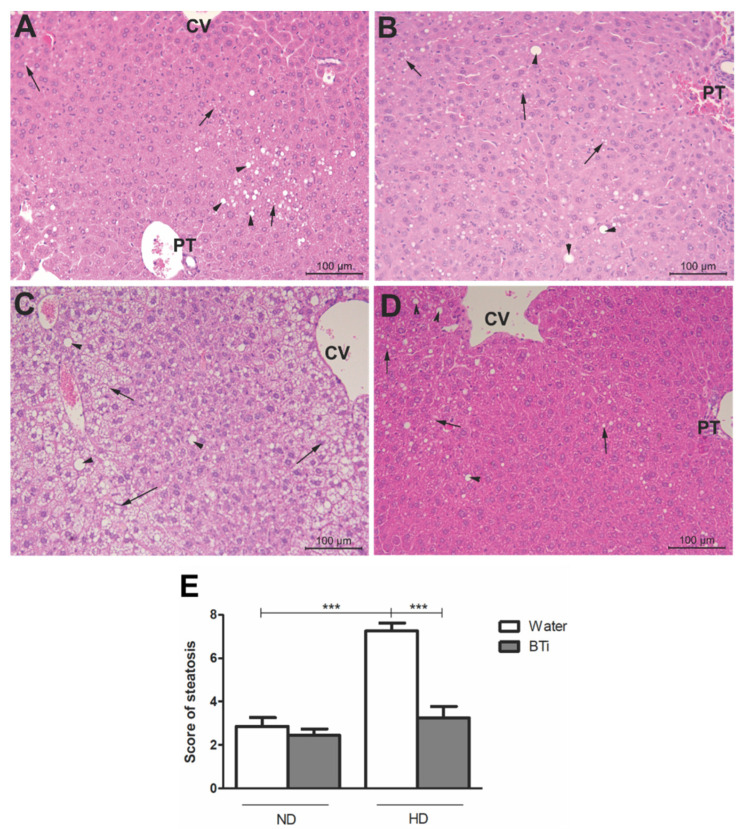
Effect of BTi treatment on liver histology. Histological sections stained with Hematoxylin-eosin. 20× magnification. (**A**) ND + water, (**B**) ND + BTi, (**C**) HD + water, (**D**) HD + BTi and (**E**) Score of steatosis. CV: Central lobular vein, PT: portal triad, Arrowhead: macrovesicular steatosis, Long arrow: microvesicular steatosis. Results expressed as median score, *n* = 5/group. Absence of lesion—2 points; mild injury—4 points; moderate injury—6 points and severe injury—8 points. ND: normolipid diet. HD: high fat diet. BTi: *B.trimera* infusion. *** *p* < 0.001. Kruskall–Wallis, followed by the Mann-Whitney post-test.

The HD induced hepatic steatosis in water-treated animals. In contrast, the BTi prevented the development of this comorbidity in the animals that ingested HD (Figure 2). The ND + water (Figure 2A) and ND + BTi (Figure 2B) groups presented normal hepatic morphological patterns without any changes. Furthermore, the HD + water group (Figure 2C) had a pattern of intense microvesicular steatosis and macrovesicular steatosis in the hepatic lobes. In contrast, the HD + BTi group (Figure 2D) presented mild, micro- and macrovesicular steatosis patterns in the hepatic lobes. Although the median hepatic steatosis of this group was mild, there was no significant difference in relation to the medians of the ND-fed groups, where steatosis was absent (Figure 2E). Both the diet and BTi did not alter the histology of the other organs evaluated (*p* > 0.05) (data not shown).

### 2.6. Effect of the BTi on Leukocyte Infiltration, Formation of Crown-like Structures by Macrophages around Adipocytes and Macrophage Cellular Functions

The number of peritoneal cells in the HD + water group was significantly higher than those in the other groups, as shown in Table 4. In addition, the BTi prevented leukocyte infiltration in the peritoneal cavity of the HD + BTi group, in comparison to the HD + water group, maintaining this parameter at a level similar to that of the ND + water group. The animals of the HD + water group had more crown-like structures (CLS) formed by macrophages around the adipocytes than the other groups fed with ND. However, the BTi prevented the formation of these structures in the HD + BTi group, maintaining an amount similar to those of the ND + water and ND + BTi groups. With regard to cellular functions, the diets and BTi treatment did not alter the cell viability of the peritoneal macrophages from animals that received the different diets (ND or HD) and treatments (water or BTi) for eight weeks. The production and release of NO was higher in the HD + water group than in both the ND groups. Furthermore, the HD + BTi group had lower nitrite levels than the HD + water group, and the level was similar to those of the ND + water and ND + BTi groups (Table 4). Neither the diets nor treatments altered the production and release of hydrogen peroxide by peritoneal macrophages.

In Table 4 we showed that regardless of diet, BTi reduced peritoneal macrophage adhesion when compared with water. With regard to cellular spreading, the macrophages from the HD + water group spread more than those from the other groups. Furthermore, BTi reduced the percentage of spread macrophages in the HD+ BTi group compared to the HD + water group, in addition to maintaining a percentage similar to those of both ND groups. As in the cell adhesion test, the macrophages of the HD + BTi group presented lower phagocytic activity than those of the HD + water group. In addition, regardless of the treatment, HD-fed animals did not have altered phagocytic activity when compared to those fed with ND.

## 3. Discussion

Infusion of the aerial parts of *B. trimera* (BTi) prevented obesity, metabolic parameters and hepatic steatosis and reduced the activity of macrophage functions, induced by the hyperlipid diet, minimizing inflammation, in the experimental model of obesity used.

The chemical profile of the BTi revealed the presence of caffeic acid derivatives, such as chlorogenic acid and its derivatives, derivates of flavonoids, such as rutin, isoschaftoside and shaftoside, and diterpenoids of the clerodane type (Table 1, see also Figure S1 Supplementary Material). This was the first study to identify the presence of vicenin 1, isoschaftosid and schaftosid in the species *B. trimera*.

In our study, we fed a 57% fat energy HD for eight weeks, which was effective for the development of obesity, metabolic disorders and intense hepatic steatosis in animals compared with feeding ND. The HD can induce obesity and metabolic disorders in rodents similar to metabolic syndrome in humans. Therefore, the model of obesity induced by diet is preferable to the models with genetic alterations, since it is necessary that the animal model presents not only the phenotype but also the pathogenesis similar to human disease. Moreover, the high fat diets associated with physical inactivity used in these models coincide with the main risk factors for obesity in humans [22].

At the same time, the animals received oral BTi daily at a dose equivalent to that recommended for a 70 kg adult. The BTi reduced the dietary intake, weight gain, adipose tissue accumulation, adiposity index and adipocyte area of HD-fed animals and reduced the feed intake of ND-fed animals, demonstrating a satiating effect of the BTi. The antiadipogenic [8] and inhibitory effects of aqueous extracts and *B. trimera* infusions on α- and β-glycosidases [23] have been described. In addition, several studies with caffeic acid and chlorogenic acid and its derivatives have reported effects related to weight loss and prevention of metabolic syndrome [24,25,26].

Daily treatment with the BTi reduced hyperglycemia, total cholesterol, LDL cholesterol and the atherogenic index, and prevented the development of insulin resistance and hepatic steatosis in HD-fed animals. The hypoglycemic effect of the *B. trimera* extract aqueous fraction was demonstrated in the literature [27]. Souza et al. (2012) [23] have demonstrated the antiobesogenic and hypocholesterolemic effects of *B. trimera* methanolic extract at different doses, in Wistar rats. In addition, studies with clerodane-type diterpenoids belonging to other plant genera [28] and with chlorogenic acid and its derivatives presented similar results, regarding weight loss, hepatic steatosis and lipid and glycemic profiles of the animals [29]. Li et al. (2009) have demonstrated that C-glycosylated flavones, such as isoschaftoside, had an inhibitory effect on α-glycosidases even greater than acarbose [30]. Thus, isoschaftoside may also contribute to the satiating effect already mentioned here, which was observed in animals treated with the BTi. Furthermore, studies suggest a moderate inhibitory effect on pancreatic lipase by rutin and isoschaftosid [31], as well as possible antiobesogenic, hypoglycemic and hypocholesterolemic effects of schaftoside [32] and rutin [33].

BTi reduced leukocyte infiltration into the peritoneal cavity in the mice, as well as the subsequent formation of crown-like structures (CLS) by macrophages around adipocytes. With adipocyte hypertrophy, blood vessels in the adipose tissue are compressed, preventing the proper supply of oxygen in the tissue and thus causing local hypoxia, which leads to adipocyte death and induces the infiltration of macrophages into the adipose tissue, resulting in the formation of crown-like structures around dead adipocytes [34]. The accumulation of macrophages in the adipose tissue is mainly responsible for the increase in inflammatory cytokines, such as TNF and IL-6, leading to the inflammation associated with excess body fat [35].

Our data have demonstrated that the HD triggered inflammation in the animals, evidenced by the infiltration of macrophages into the adipose tissue associated with the formation of CLS around the adipocytes, increased production and release of NO_2_^−^ and spreading of peritoneal macrophages. In the mice group submitted to HD, the BTi treatment was effective in preventing inflammation. We observed that the macrophages spreading, adhesion, phagocytic activity and nitric oxide production was reduced in the mice that received BTi treatment. However, the release of H_2_O_2_ did not reduce.

Hyperlipidic diets induce changes in the composition of the intestinal microbiota that favor the transfer of endogenous LPS from the gut lumen into the bloodstream, leading to metabolic endotoxemia [36]. LPS binding to TLR-4 triggers an inflammatory cascade activated by the NF-κB signaling pathway, which promotes the transcription of several proinflammatory genes and enzymes, such as iNOS [37]. Increase of iNOS expression, which is involved in the production of reactive nitrogen species, such as NO, is increased in obese individuals [38]. Thus, the reduction of NO production in mice submitted to HD and treated with BTi may be contributing to reduced activation of macrophages and other inflammatory cells. The antioxidant effect of *B. trimera* was described [8]. In addition, related to the genus *Baccharis*, there are reports in the literature that point out the antioxidant effect of chlorogenic acid and its derivatives on humans and animals [39]. Gao, Ma and Liu (2013) studied the effects of rutin on inflammation in RAW 264.7 macrophage culture and on obesity and inflammation in an experimental model of obesity in C57BL/6 mice fed-HD for eight weeks. The authors observed that in vitro treatment with rutin did not affect the macrophages viability but suppressed the production of TNF. However, in vivo treatment with this flavonoid prevented weight gain and decreased adipocyte area and the formation of CLS by macrophages around adipocytes, hepatic steatosis and insulin resistance, as well as reduced the mRNA levels of MCP-1 and TNF in the adipose tissue.

Thus, we suggest that the preventive effect of the BTi on obesity, insulin resistance, hepatic steatosis and inflammation in our experimental model is due to the possible inhibition of the NO production and macrophage activation. However, the reduction of other pro-inflammatory mediators cannot be ruled out. In addition, we suggest that the effects presented here are due to the presence of phenolic compounds in the BTi, such as chlorogenic acid and its derivatives and flavonoids, such as rutin.

## 4. Materials and Methods

### 4.1. Plant Material

*Baccharis trimera* was obtained from Flor do Campo Company^®^ (Rio do Campo, SC, Brazil, batch C0719062) and the aerial parts were used to prepare the *B. trimera* infusion (BTi). It was identified as *B. trimera* (Less.) DC by Flavio Macedo Alves, based on the comparison with herbarium samples, a specialized bibliography and the type specimens. A voucher specimen was deposited in the CGMS herbarium at the Federal University of Mato Grosso do Sul (number 75817).

### 4.2. Preparation of the BTi

The plant material was sprayed, and the sample was prepared by infusion, as indicated by the National Agency for Sanitary Surveillance [2], at a dose of 7.5 g/day (for an individual of 70 kg), which is equivalent to about 100 mg/kg/day. Considering the obtained yield (14.1% *w*/*w*), the dose used was 15 mg/kg/day. The material was filtered, lyophilized and stored at 2–8 °C until use.

### 4.3. Analysis of the BTi by HPLC-DAD-MS/MS

HPLC coupled to a detector was performed by a diode array and mass spectrometer (HPLC-DAD-MS/MS) to verify the presence of the main compounds present in the commercial sample of *B. trimera* that was acquired. Two microliters (of a solution at 1 mg/mL prepared with ethanol and water 7:3 *v*/*v*) of BTi were injected into a UFLC LC-20AD (Shimadzu) coupled to a diode array detector (DAD) and a mass spectrometer microTOF-Q III (BrukerDaltonics^®^) that provided an electrospray ionization source and analyzers for quadrupole—time-of-flight (qTOF). The C-18 column (Kinetex^®^, 2.6 μm, 150 × 2.2 mm) was protected by a precolumn packed with the same material. The mobile phase was a mixture of water (solvent A) and acetonitrile (solvent B), both containing 1% acetic acid (*v*/*v*) with a gradient elution profile of 0–2 min, 3% of B; 2–25 min, 3–25% of B; and 25–40 min, 25–80% of B. The elution was followed by column washing and reconditioning (8 min). The flow rate was 0.3 mL/min, and the column was maintained at 50 °C. The analysis was monitored at wavelengths of 240–800 nm, and the mass spectrophotometer operated in the negative and positive ionization mode (*m*/*z* 120–1200).

Compound identification was carried out with ultraviolet (UV) spectra, accurate mass, and ESI fragmentation patterns compared to those previously published in the literature [40,41,42]. Compounds were compared to the standard described for the genus *Baccharis* or species *B. trimera* or were identified by comparison with the authentic standard (quinic acid (77-95-2), caffeine quercetin (1241-87-8), rutin (153-18-4), 3,4-dicaffeoylquinic acid (57378-72-0), 3,5-dicaffeoylquinic acid (89919-62-0), and 4,5-dicaffeoylquinic acid (89886-31-7). All reagents were obtained from Sigma-Aldrich^®^ (St. Louis, Missouri, EUA).

### 4.4. Animals and Diet

Swiss male mice, 5 weeks old from the UFMS Central Warehouse (Campo Grande, MS, Brazil) were kept in cages at 22 ± 2 °C with a light/dark cycle of 12 h and received a standard diet (Nuvital^®^) and water at will for a week of adaptation. After, the animals were fed ad libitum with water and diet for adult mice, as standardized by the American Institute of Nutrition (AIN-93M) [43]. The normolipid diet (ND) contained 75.81% carbohydrate (*w*/*w*) and 9.47% lipid (*w*/*w*). The hyperlipidic diet (HD) contained 31.93% carbohydrates (*w*/*w*) and 57.13% lipids (*w*/*w*) (Table S1 Supplementary Material). The diets were purchased from PragSoluções Biociências^®^ (Jaú, SP, Brazil) and kept at 2 to 8 °C until use. The experiments were conducted according to the CONCEA (National Council for Control of Animal Experimentation) and approved by the Committee on Ethics in the Use of Animals—CEUA/UFMS (Protocol number 770/2016; 15 June 2016).

### 4.5. Experimental Design

The study was conducted after eight weeks of induction of obesity and treatment with BTi. The animals were randomly assigned to one of four groups: ND + water (10 mL/kg/day, oral (v.o.), gavage); ND + BTi (15 mg/kg/day, v.o., gavage); HD + water (10 mL/kg/day, v.o., gavage) and HD + BTi (15 mg/kg/day, v.o., gavage). Weight gain and food intake were measured three times a week. At the end of the experiment, the mice were euthanized by cervical dislocation. The organs, visceral fat and blood were collected and referred for analysis.

### 4.6. Measurement of Biochemical Parameters

Insulin sensitivity was assessed four days before euthanasia, and the animals were fed at the moment of caudal blood collection. The determination of fasting glucose and the oral glucose tolerance test were performed on the day of euthanasia, with animals fasting for 8 h prior. In both, glycemia was measured by means of a glucometer and reactive strips (Accu-Chek Performa^®^). Serum concentrations of total cholesterol (TC), triglycerides (TG), and HDL-cholesterol (HDL-c) were measured using enzyme kits (LabTest^®^, Lagoa Santa, MG, Brazil). The levels of VLDL and LDL cholesterol (LDL-c) and the atherogenic index were calculated from the values of CT, TG and HDL-c, according to Equations (1)–(3), respectively:

(1) $$\mathrm{VLDL}\;(\mathrm{mg}/\mathrm{dL})=\frac{\mathrm{TG}}{5}$$

LDL (mg/dL) = TC − (HDL + VLDL)(2)

(3) $$\mathrm{Atherogenic}\;\mathrm{index}=\frac{\mathrm{TC}}{\mathrm{HDL}}$$

### 4.7. Histological Analysis

The heart, kidney, duodenum, pancreas, liver and epididymal, retroperitoneal, perirenal, mesenteric and omental adipose cushions were collected and weighed after euthanasia, and the organs and epididymal fat were processed for histological analysis according to previously described [44]. The tissues were embedded in paraffin, sectioned at 5 μm and stained with hematoxylin-eosin. A microscope (Primo Star, Zeiss^®^) coupled to a digital camera (Nikon D3100^®^) with a final 20 to 100× magnification was used to obtain photos. The images were analyzed using software Motic Images Plus 2.0 (Motic China Group Co., Ltd.; Hong Kong, China).

### 4.8. Peritoneal Macrophages Harvesting and Counting

The peritoneal cells of animals that received different diets (ND or HD) and treatments were collected and plated in 96-well microplates. Assays were performed without further stimulation, except for the H_2_O_2_ determination. These cells were harvested by washing the cavities with five milliliters of sterile phosphate-buffered saline (PBS) containing heparin (10 U/mL). The total count of peritoneal leukocytes was performed in a Neubauer chamber, and the results are expressed as the number of cells/mm^3^. Then, the peritoneal exudate was centrifuged (1000 rpm/5 min), and the cell pellet was resuspended in supplemented RPMI 1640 medium (Sigma-Aldrich^®^) (10% fetal bovine serum (FBS), penicillin (100 U/mL), streptomycin (100 mg/mL) and 2 mM glutamine (Sigma-Aldrich^®^).

### 4.9. MTT Assay for Cell Viability

Cell viability was evaluated as described by Mosmann (1983) [45]. This assay was performed together with the nitric oxide (NO) assay. MTT [3-(4,5-dimethylthiazol-2-yl)-2,5-diphenyltetrazolium bromide, 20 µL, 5 mg/mL, Sigma-Aldrich^®^) was added to each well, and the cells were incubated for an additional 2 h. The medium was discarded, and the formazan that formed in the cells was dissolved in DMSO (200 µL, Sigma-Aldrich^®^). The absorbance was determined using an ELISA reader (Humareader HS^®^) at a wavelength of 540 nm. The results are expressed as the percentage of viable cells.

### 4.10. Nitric Oxide Production by Peritoneal Macrophages

NO production was determined by the Griess method [46]. The peritoneal cells were dispensed into a 96-well plate (1 × 10^5^ cells/well) and incubated with 100 μL supplemented RPMI 1640 medium at 37 °C in a 5% CO_2_ atmosphere. After 24 h, the wells were washed three times with phosphate-buffered saline (PBS) for the removal of the non-adherent cells. Then, the adhered cells (macrophages) were incubated for 48 h with 100 μL supplemented RPMI 1640 medium. At the end of this period, the absorbance was determined using an ELISA reader at a wavelength of 540 nm. Nitrite concentration values were expressed as µM NO_2_^−^.

### 4.11. Hydrogen Peroxide Production by Peritoneal Macrophages

The production of hydrogen peroxide (H_2_O_2_) was as described by Pick and Mizel (1981) [47]. A total of 1 × 10^5^ cells/well was resuspended in supplemented RPMI 1640 medium and incubated for 1 h in a 96-well plate to allow adhesion. Subsequently, the non-adherent cells were removed, and 100 µL phenol red solution (140 mM NaCl; 10 mM potassium phosphate buffer, pH 7.0; 5.5 mM dextrose; and 0.56 mM phenol red) containing 8.5 U/mL horseradish peroxidase (Sigma-Aldrich^®^) in the presence or absence of 10 µL phorbol myristate acetate (PMA, 100 ng/mL, Sigma-Aldrich^®^) was added, and the cells were incubated for 1 h at 37 °C in a 5% CO_2_ atmosphere. The plate was incubated for another 1 h, and the reaction was stopped by the addition of 10 µL 1 N NaOH. The absorbance was determined using an ELISA reader at a wavelength of 620 nm. Hydrogen peroxide concentration values were expressed as µM H_2_O_2_.

### 4.12. Adhesion and Spreading of Peritoneal Macrophages

Cellular adhesion capacity was determined by the crystal violet method as described by Santos et al. (2016) [48]. For this assay, the cells were dispensed at a concentration of 1 × 10^5^ cells/well into 96-well plates and incubated with 100 μL supplemented RPMI medium for 2 h under the above conditions. After the incubation period, the culture medium was removed, and the cells were fixed with 30 μL of 4% paraformaldehyde for 10 min at room temperature. After fixation, 50 μL crystal violet solution in methanol (2.5% *v*/*v*) was added to each well. The plate was held for 30 min in the dark at room temperature. Then, the cells were washed with PBS, and 50 μL 0.1 M sodium citrate pH 4.2 was added to each well. The supernatant was transferred to another plate, and the absorbance was determined at a wavelength of 540 nm with an ELISA reader. The results are expressed as Absorbance/1 × 10^5^ cells.

Spreading assays were performed according to the method described by Rabinovitch and Destefano (1973) [49]. Peritoneal cells (1 × 10^5^ cells/well) were placed into a 24-well plate containing a circular glass coverslip in each well. Cells were incubated with 600 μL supplemented RPMI 1640 medium for 2 h under the above conditions, and the cells that adhered to the coverslip were fixed in 2.5% glutaraldehyde and examined with a phase contrast microscope (Optiphase^®^). One hundred cells were counted and scored as round or spread. An index of macrophage spreading (SI) was calculated as (number of spread macrophages × 100)/100, SI = % of spread macrophages.

### 4.13. Phagocytic Activity of Peritoneal Macrophages

A suspension of zymosan (Sigma-Aldrich^®^; 5 mg/mL in PBS) was opsonized with homologous serum obtained from normal mice according to a method previously described [50]. The cell suspension was adjusted to 1 × 10^6^ cells/100 μL RPMI 1640 medium containing 5 mM glucose and 2% albumin and kept in an ice bath until assayed. In the assays, the cell suspension and zymosan suspension (1:1 *v*/*v*) or RPMI (control) were added to plastic tubes and incubated for 30 min at 37 °C under the above conditions. Aliquots were collected, scattered on glass slides and stained with HEMA^3^. The activity was evaluated by counting 100 cells that had phagocytosed at least one zymosan particle.

### 4.14. Statistical Analysis

The results were expressed as mean ± standard error of the mean (SEM). To determine the statistical significance level, ANOVA was used followed by Tukey post-test (parametric data) and Kruskal–Wallis (non-parametric data) followed by the Mann–Whitney post-test. Values less than 5% (*p* < 0.05) were considered significant using GraphPad Prism^®^ 5.0 software (GraphPad; San Diego, CA, USA).

## 5. Conclusions

The daily intake of the infusion produced from the aerial parts of *B. trimera*, according to the dosage and method of preparation recommended by regulators for human use, can help to fight obesity and its metabolic alterations, as it is able to prevent obesity, metabolic disorders, insulin resistance, hepatic steatosis and subclinical inflammation, validating the popular use of this plant infusion/tea.

## Figures and Tables

**Table 1:** Compounds identified by HPLC-DAD-MS/MS in the *Baccharis trimera* infusion (BTi).

Peak	Compound (CAS)	Ident. Level ^a^	RT (min)	UV (nm)	[M + H]^+^ *m*/*z*	[M − H]^−^ *m*/*z*	Molecular Formula	MS/MS (Negative Mode) *m*/*z*
1	quinic acid (77-95-2)	1	1.2	-	193.0707	191.0561	C_7_H_12_O_6_	-
2	caffeoylquinic acid (1241-87-8)	1	10.6	217–328	355.1024	353.0878	C_16_H_18_O_9_	191(C_7_H_11_O_6_)^−^
3	5-feruloyl-quinic acid	2	11.0	215–322	369.1552	367.1035	C_17_H_20_O_9_	191(C_7_H_11_O_6_)^−^
4	Vicenin 1	2	16.1	216–340	565.1552	563.1406	C_26_H_28_O_14_	503(C_24_H_23_O_12_)^−^, 473(C_23_H_21_O_11_)^−^, 443(C_22_H_19_O_10_)^−^, 425(C_22_H_17_O_9_)^−^, 413(C_25_H_17_O_6_)^−^, 383(C_20_H_15_O_8_)^−^, 353(C_19_H_13_O_7_)^−^, 297(C_17_H_13_O_5_)^−^
5	isoschaftoside	2	16.6	216–340	565.1552	563.1406	C_26_H_28_O_14_	545(C_26_H_25_O_13_)^−^, 503(C_24_H_23_O_12_)^−^, 473(C_23_H_21_O_11_)^−^, 443(C_22_H_19_O_10_)^−^, 425(C_22_H_17_O_9_)^−^, 413(C_21_H_17_O_9_)^−^, 383(C_20_H_15_O_8_)^−^, 353(C_19_H_13_O_7_)^−^, 297(C_17_H_13_O_5_)^−^
6	schaftoside	2	17.7	217–340	565.1552	563.14.06	C_26_H_28_O_14_	473(C_23_H_21_O_11_)^−^, 443(C_22_H_19_O_10_)^−^, 425(C_22_H_17_O_9_)^−^, 413(C_21_H_17_O_9_)^−^, 383(C_20_H_15_O_8_)^−^, 353(C_19_H_13_O_7_)^−^, 297(C_17_H_13_O_5_)^−^
7	rutin (153-18-4)	1	18.6	279–350	607.1607	609.1461	C_27_H_30_O_16_	301(C_15_H_9_O_7_)^−^, 271(C_14_H_7_O_6_)^−^, 255(C_14_H_7_O_5_)^−^
8	3,4-dicaffeoylquinic acid (57378-72-0)	1	20.0	217–325	517.1341	515.1195	C_25_H_24_O_12_	191(C_7_H_11_O_6_)^−^, 179(C_9_H_7_O_4_)^−^, 173(C_7_H_9_O_5_)^−^, 161(C_9_H_7_O_4_)^−^
9	3,5-dicaffeoylquinic acid (89919-62-0)	1	20.6	218–325	517.1341	515.1195	C_25_H_24_O_12_	191(C_7_H_11_O_6_)^−^, 179(C_9_H_7_O_4_)^−^, 161(C_9_H_7_O_4_)^−^
10	4,5-dicaffeoylquinic acid (89886-31-7)	1	22.2	219–325	517.1341	515.11995	C_25_H_24_O_12_	191(C_7_H_11_O_6_)^−^, 179(C_9_H_7_O_4_)^−^, 173(C_7_H_9_O_5_)^−^
11	putative clerodane diterpenoid	3	28.5	-	347.1856	391.1762 *	C_20_H_26_O_5_	345(C_20_H_25_O_5_)^−^, 217(C_14_H_17_O_2_)^−^
12	putative clerodane diterpenoid	3	29.9	-	349.2005	393.1919 *	C_20_H_28_O_5_	-
13	Putative hexosyl-coumaroyl-triterpene	3	30.8	310	843.4161	420.2009 **	C_45_H_62_O_15_	679(C_36_H_55_O_12_)^−^, 599(C_31_H_51_O_11_)^−^, 179(C_9_H_7_O_4_)^−^, 161(C_9_H_5_O_3_)^−^
14	Unknown	3	31.3	287–325	-	441.2041 **	C_47_H_64_O_16_	-
15	dihydroxy-trimethoxyflavone	3	32.6	276–339	345.0969	343.0822	C_18_H_16_O_7_	313(C_16_H_9_O_7_)^−^, 285(C_15_H_9_O_6_)^−^

^a^: Identification level for metabolites according to Metabolomics Standards Initiative (MSI). Levels: (1) identified metabolites by comparison with standard, (2) putatively annotated compounds, (3) putatively characterized compound classes and (4) unknown compounds. RT: retention time; mass errors and mSigma below 5 ppm and 30, respectively. * [M + HCOOH-H]^−^, ** [2M-H]^−.^

**Table 2:** Effect of the BTi on food and caloric daily intake, weight gain, liver and fat pad weights, adiposity index and adipocyte area.

Parameter	ND		HD	
	Water	BTi	Water	BTi
Food intake (g)	4.22 ± 0.03	3.75 ± 0.06 ***	3.41 ± 0.05 ***	3.04 ± 0.04 ^###^
Calorie intake (kj)	67.07 ± 0.54	59.66 ± 0.92 ***	73.05 ± 1.05 ***	65.06 ± 0.75 ^###^
Initial body weight (g)	32.62 ± 0.76	30.28 ± 0.84	30.89 ± 1.16	30.33 ± 1.07
Final body weight (g)	48.03 ± 0.90	41.14 ± 1.41 *	55.71 ± 1.24 *	47.22 ± 1.84 ^##^
Weight gain (g)	14.76 ± 0.83	10.86 ± 0.86 *	24.82 ± 1.19 ***	16.89 ± 0.94 ^###^
Omental (g)	0.05 ± 0.01	0.07 ± 0.01	0.07 ± 0.01	0.09 ± 0.01 *
Retroperitoneal (g)	1.26 ± 0.13	0.65 ± 0.07	1.76 ± 0.12 *	1.13 ± 0.08 ^###^
Epididymal (g)	1.98 ± 0.07	1.57 ± 0.09	2.89 ± 0.20 ***	2.41 ± 0.13
Mesenteric (g)	1.08 ± 0.05	0.79 ± 0.05	2.21 ± 0.15 ***	1.26 ± 0.11 ^###^
Perirenal (g)	0.28 ± 0.02	0.23 ± 0.02	0.45 ± 0.06 *	0.35 ± 0.04
Visceral adipose tissue (g)	4.15 ± 0.48	3.30 ± 0.19	7.39 ± 0.43 ***	5.24 ± 0.22 ^###^
Adiposity index (%)	9.67 ± 0.36	7.94 ± 0.26 *	13.25 ± 0.62 ***	11.09 ± 0.29 ^##^
Adipocyte area (µm^2^)	580.1 ± 6.6	525.3 ± 5.4 ***	740.3 ± 6.4 ***	695.1 ± 7.8 ^###^
Liver (g)	2.00 ± 0.12	1.79 ± 0.10	2.69 ± 0.20 *	1.95 ± 0.15 ^##^

Results represent mean ± S.E.M. *n* = 8/group. ND: normolipid diet. HD: high-fat diet. BTi: infusion of *B. trimera*. * *p* < 0.05; *** *p* < 0.001 compared to ND + water group; ^##^ *p* < 0.01 and ^###^ *p* < 0.001 compared to HD + water group. ANOVA, followed by the Tukey post-test.

**Table 3:** Effect of the BTi on the mice lipid profile.

Parameter	ND		HD	
	Water	BTi	Water	BTi
TG (mg/dL)	149.7 ± 14.2	121.3 ± 11.9	142.4 ± 7.3	149.7 ± 8.5
TC (mg/dL)	160.0 ± 17.3	131.9 ± 10.0	218.6 ± 5.9 **	164.6 ± 9.9 ^##^
HDL-c (mg/dL)	90.0 ± 9.7	80.9 ± 6.4	93.0 ± 3.4	95.3 ± 4.6
LDL-c (mg/dL)	40.0 ± 8.4	26.7 ± 3.5	97.1 ± 4.0 ***	39.3 ± 5.8 ^###^
VLDL-c (mg/dL)	30.0 ± 2.8	24.3 ± 2.4	28.5 ± 1.5	30.0 ± 1.7
AI	1.8 ±0.1	1.7 ± 0.0	2.4 ± 0.1 ***	1.7 ± 0.0 ^###^

Results represents mean ± S.E.M. *n* = 6/group. ND: normolipid diet. HD: high-fat diet. BTi: infusion of *B. trimera*. TG: triglycerides. TC: total cholesterol. HDL-c: high-density lipoprotein cholesterol. LDL-c: low-density lipoprotein cholesterol. VLDL: very-low-density lipoprotein cholesterol. AI: atherogenic index. ** *p* < 0.01 and *** *p* < 0.001 compared to ND + water group; ^##^ *p* < 0.01 and ^###^ *p* < 0.001 compared to HD + water group. ANOVA, followed by the Tukey post-test.

**Table 4:** Effect of BTi on leukocyte infiltrate, formation of crown-like structures (CLS) by macrophages, around adipocytes, and cellular functions of macrophages.

Parameter	ND		HD	
	Water	BTi	Water	BTi
Leukocyte infiltrate (cell/mm^3^)	1540 ± 95	1531 ± 132	2280 ± 127 **	1481 ± 81 ^###^
CLS/field	0.82 ± 0.20	0.76 ± 0.12 ***	4.20 ± 0.61 ***	1.36 ± 0.30 ^###^
Cell viability (%)	97.66 ± 1.59	89.38 ± 2.82	92.13 ± 3.01	93.42 ± 1.78
Nitric oxide (µM NO_2_^−^)	7.09 ± 1.61	10.41 ± 0.10	34.12 ± 4.34 ***	8.36 ± 1.14 ^###^
Hydrogen peroxide (µM H_2_O_2_)	28.16 ± 1.61	34.09 ± 2.60	28.60 ± 2.32	34.56 ± 2.21
Spreading (%)	10.47 ± 0.83	11.37 ± 0.69	25.00 ± 4.96 ***	9.79 ± 0.96 ^###^
Cell adhesion (abs/1 × 10^5^ cell)	0.42 ± 0.02	0.28 ± 0.02 ***	0.43 ± 0.03	0.25 ± 0.01 ^###^
Phagocytosis (%)	48.00 ± 2.31	37.75 ± 1.32	55.00 ± 3.79	32.67 ± 2.33 ^###^

Results represents mean ± S.E.M., *n* = 6/group. ND: normolipid diet. HD: high-fat diet. BTi: infusion of *B. trimera*. ** *p* < 0.01 and *** *p* < 0.001 compared to ND + water group; ^###^ *p* < 0.001 compared to HD + water group. ANOVA, followed by Tukey post-test.

## Data Availability

Data is contained within the article and supplementary material.

## Supplementary Materials

The following supporting information can be downloaded at: https://www.mdpi.com/article/10.3390/ph15101258/s1, Figure S1: Chromatographic profile of the infusion of *Baccharis trimera*; Table S1: Composition of the experimental diets.

## Sample Availability

Samples of the compounds are available from the authors.
