# Supplementary material for: Baccharis trimera Infusion Reduces Macrophages Activation and High-Fat Diet-Induced Metabolic Disorders in Mice
Source: Pharmaceuticals (Basel). 2022 Oct 13;15(10):1258. doi: 10.3390/ph15101258 (PMC9611608; doi:10.3390/ph15101258)
Supplement: Supplementary file 1 [file pharmaceuticals-15-01258-s001.zip › pharmaceuticals-1882286-supplementary.pdf]

pharmaceuticals

MDPI

SUPPLEMENTARY MATERIAL

Baccharis trimera Infusion Reduces Macrophages Activation and High-Fat Diet-Induced Metabolic Disorders in Mice

Thalita Vieira Nascimento Ximenes [1], Raquel Carvalho [1], Iluska Senna Bonfá [1], Vanessa Samúdio Santos [1], Luciane Candeloro [2], Flávio Macedo Alves [2], Denise Brentan Silva [1], Carlos Alexandre Carollo [1], Karine de Cássia Freitas Gielow [1], Saulo Euclides Silva-Filho [1] and Mônica Cristina Toffoli-Kadri [1*]

[1] Pharmaceutical Sciences, Food and Nutrition College, Federal University of Mato Grosso do Sul, Campo Grande 79070-900, Brazil
[2] Biosciences Institute, Federal University of Mato Grosso do Sul, Campo Grande 79070-900, Brazil
* Correspondence: monica.kadri@ufms.br

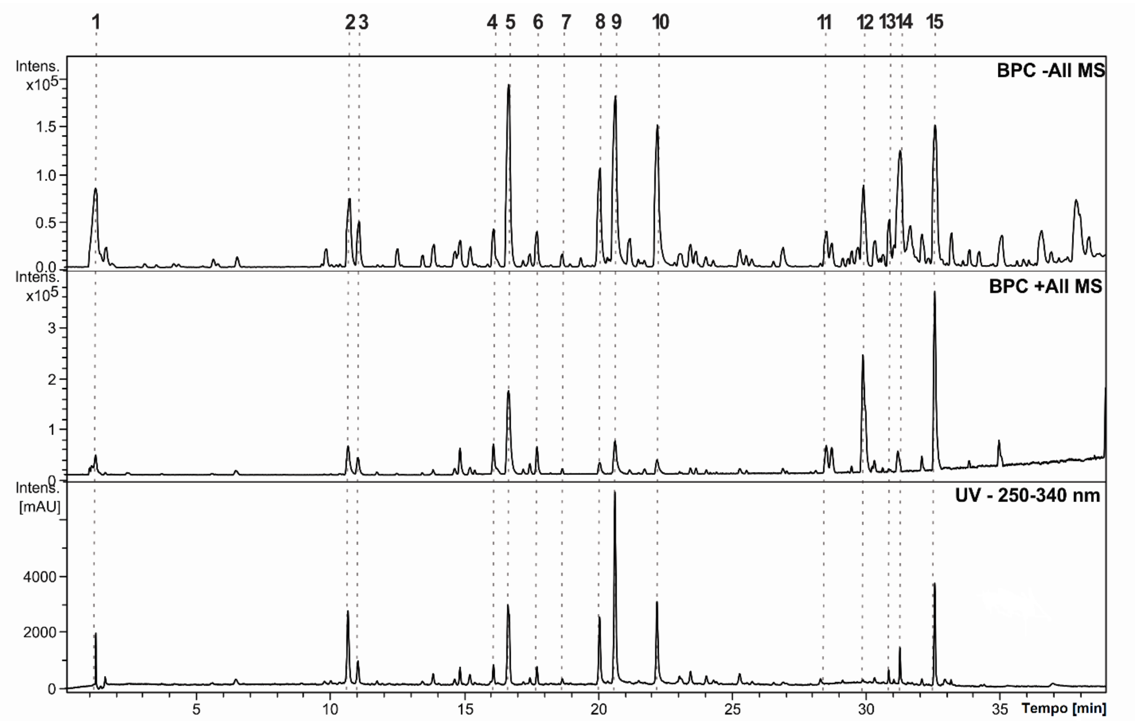

Figure S1. Chromatographic profile of the infusion of *Baccharis trimera*.

Table S1. Composition of the experimental diets.

	Diet AIN-93M (Normolipid)	High-fat Diet
**Ingredients (g / kg)**		
Starch	620.7	308.7
Casein	140	140
Sucrose	100	100
Soybean oil	40	40
Cellulose	50	50
Mineral Mix AIN 93	35	35
Vitamin Mix AIN 93	10	10
L-Cystine	1.8	1.8
Choline	2.5	2.5
BHT	0.008	0.008
Lard	-	312.0
**Energy (Kj / kg)**	15940.9	21421.2
Carbohydrates (%)	75.81	31.93
Proteins (%)	14.73	10.94
Lipids (%)	9.47	57.13
**Kj / g**	15.90	21.42
